# The impact of prenatal mental health on birth outcomes before and during the COVID-19 pandemic in Anhui, China

**DOI:** 10.1371/journal.pone.0308327

**Published:** 2024-08-06

**Authors:** Tianqi Zhao, Gian S. Jhangri, Keith S. Dobson, Jessica Yijia Li, Shahirose S. Premji, Fangbiao Tao, Beibei Zhu, Shelby S. Yamamoto

**Affiliations:** 1 School of Public Health, University of Alberta, Edmonton, AB, Canada; 2 Faculty of Arts, Department of Psychology, University of Calgary, Calgary, AB, Canada; 3 Faculty of Social Sciences, Department of Psychology, University of Victoria, Victoria, BC, Canada; 4 Faculty of Health Sciences, School of Nursing, Queen’s University, Kingston, Ontario, Canada; 5 Department of Maternal, Child and Adolescent Health, School of Public Health, Anhui Medical University, Hefei, China; University of Zurich, SWITZERLAND

## Abstract

Adverse birth outcomes remain challenging public health problems in China. Increasing evidence indicated that prenatal depression and anxiety are associated with adverse birth outcomes, highlighting the importance and severity of prenatal depression and anxiety in China. The COVID-19 pandemic is likely to further exacerbate prenatal mental health problems and increase the risk of adverse birth outcomes. The aim of this study is to assess and compare the impacts of prenatal mental health issues on birth outcomes before and during the COVID-19 pandemic in Ma’anshan, Anhui, China. Participants in this study were women who visited local maternal and child health hospitals in Ma’anshan, Anhui, China. Two independent sets of individual maternal data (n_pre-pamdemic_ = 1148; n_pandemic_ = 2249) were collected. Prenatal depression and anxiety were measured online using the Edinburgh Postnatal Depression Scale (EPDS) and the General Anxiety Disorder-7 (GAD-7). Adverse birth outcomes were determined using hospital-recorded infant birth weight and gestational age at delivery. In this study, we found that the pandemic cohort had lower mean EPDS and GAD-7 scores than the pre-pandemic cohort. The prevalence of prenatal depression (14.5%) and anxiety (26.7%) among the pandemic cohort were lower than the pre-pandemic cohort (18.6% and 36.3%). No significant difference was found in the prevalence of adverse birth outcomes comparing the two cohorts. Prenatal depression was associated with small gestational age only in the pandemic cohort (OR = 1.09, 95% CI 1.00–1.19, p = 0.042). Overall, this study highlighted an association between prenatal depression and small for gestational age in Anhui, China. Addressing prenatal depression may thus be key in improving birth outcomes. Future studies could focus on potential causal relationships.

## Introduction

Pregnancy is a critical time that precedes motherhood for many women. Women face a multitude of different biological and psychological changes and contend with changes in familial and societal social status [1]. These changes can be overwhelming and lead to some types of mental health symptoms and disorders [1]. China has a high rate of prenatal depression and anxiety compared to other countries [2]. Studies have shown the prevalence of prenatal depression in China to be between 3.6% to 40.2%, and prenatal anxiety to be between 1.8% to 42.1%,compared to the global average of 18.2% to 24.6% for prenatal depression and 7.4% to 12.8% for prenatal anxiety [1,3].

Disasters and infectious disease outbreaks have been known to increase the risk of developing depression and anxiety [4,5]. Thus, with the significant threats from the coronavirus disease 2019 (COVID-19) pandemic, pregnant women could be at an even higher risk of developing prenatal depression and anxiety [4,5]. In China, some studies suggested that the rates of prenatal mental health issues have increased since the declaration of the pandemic. For example, Dong et al. suggested that the level of depression among pregnant women has significantly increased during the COVID-19 pandemic (50.6%) compared to the period before the pandemic (3.5% to 8.2%), with nearly 27% of the pregnant women experiencing moderate to severe depression [6]. Another study reported the estimated prevalence of prenatal anxiety increased from 15.2% pre-pandemic to 37.0% during the pandemic [7]. Anhui has the highest prevalence of perinatal depression (33%) in mainland China, according to a meta-analysis that examined 95 studies across 23 Chinese regions [3,8].

Prenatal depression and anxiety have been found to be associated with adverse birth outcomes, including preterm birth, low birth weight and small for gestational age [1,9]. Adverse birth outcomes are the leading causes of neonatal deaths and under-five years mortality and serve as key risk factors for the development of neurological damage, respiratory diseases, visual and hearing impairment, later-life morbidities [10–12]. Using the psychobiobehavioral model, researchers have hypothesized that complex psychological, biological, and behavioral factors explain the association between adverse birth outcomes and their associated morbidities [13]. A stressful event, such as the pandemic, could trigger an increased risk of developing adverse birth outcomes via stress pathways [14,15].

To our knowledge, there has been no original research exploring the impacts of prenatal depression and anxiety on birth outcomes before and during the pandemic in China [16]. This epidemiological study aims to explore the association between prenatal mental health issues and birth outcomes and highlight key findings concerning the psychosocial impacts of pregnancy and related risk factors before and during the COVID-19 pandemic, to help mitigate adverse birth outcomes in other public health emergencies.

## Methodology

### Study setting, population, and data collection

This study was conducted in the city of Ma’anshan, Anhui province, China. Anhui is a landlocked province in the East China region, bordered by Hubei province which is the epicenter of the COVID-19 pandemic in China [17,18]. As of March 22, 2023, Anhui has reported more than 70,000 cases [19].

Participants were women aged of 18 years and above who visited local maternal and child health hospitals in Ma’anshan. Maternal data were collected at two timepoints, from different cohorts: 1) Pre-pandemic data (n = 1148) were collected between May 27 and September 11, 2019; and 2) Pandemic data (n = 2249) were collected between April 27 and August 20, 2020. The data collection process was carried out by a team trained in the use of the tools, and followed data collection procedures and protocols [8]. Sociodemographic, prenatal depression, and anxiety measures were administered using online self-report questionnaires. Birth outcome measures were collected from existing medical records. Medical records were retrieved and entered after participants’ deliveries. Pre-pandemic cohort medical records were accessed and entered between July 27, 2020, and May 11, 2021. Pandemic cohort medical records were accessed and entered between October 13, 2020, and April 25, 2021. Identifiable information was removed upon data cleaning. Pre-pandemic baseline data were collected as part of a larger project focused on implementing a prenatal depression screening and management program, Mom’s Good Mood, in Anhui, China [8]. Written informed consent forms were provided to and signed by participants. Data were stored on a secure server.

### Measurements

#### Socio-demographic information

Demographic information was collected on participants’ age, body weight before pregnancy, body mass index (BMI) before pregnancy, number of people in the household, self-reported economic status, ethnicity, residential area, marital status, household income, education, employment, smoking, passive smoking exposure, alcohol consumption, attitudes toward current pregnancy, how current pregnancy occurred, history of adverse birth outcomes, history of mental health diagnoses, history of drug-use, multigravida, and multiparous.

#### Prenatal depression

Prenatal depression was evaluated using the Edinburgh Postnatal Depression Scale (EPDS). The EPDS is a 10-item self-report questionnaire that measures emotional and behavioral symptoms, potentially indicative of depression, over the last 7 days. Each question has four options, with a range of 0 (lowest) to 3 (highest) points based on the severity of the symptom. Item 3, and items 5 to 10, are reverse-scored (0 highest, 3 lowest). The EPDS has been validated in China, with a cut-off value of 9 reflecting the presence of depressive symptomatology in the Chinese population (sensitivity: 80.0%; specificity: 83.0%) [20,21]. In this current study, participants with EPDS scores ≥9 were classified as having symptoms of depression. The severity of depression was classified by participants’ total scores: no depression (0–8), mild depression (9–11), moderate depression (12–13), and severe depression (≥14) [22,23].

#### Prenatal anxiety

Prenatal anxiety was evaluated by General Anxiety Disorder-7 (GAD-7) via online questionnaires. GAD-7 is a screening tool to assess patients’ anxiety symptoms for the past two weeks [24]. It has been confirmed to be a reliable tool to assess anxiety among the Chinese population and pregnant women [25,26]. Seven items with typical anxiety symptoms over the past 2 weeks were measured on a 4-point Likert-type scale: 0 = never, 1 = several days, 2 = more than half of the days, and 3 = nearly every day. A cut-off score of 5 or higher was considered to indicate anxiety [27]. In this study, the severity of anxiety was classified as follows: mild (5–9), moderate (10–14), and severe (15–21).

#### Birth outcomes

The main birth outcome measures were birth weight (grams) and gestational age (weeks) at delivery. Small for gestational age infants are those born with a weight that is below the 10th percentile for infants of the same gestational age [28]. In this study, small for gestational age was defined based on the newly updated growth standard curves of birth weight, length, and head circumference of Chinese newborns of different gestational ages [29]. Boys and girls were calculated separately based on their gestational age and the 10th percentile reference weights.

### Statistical analysis

Continuous variables are presented as means and standard deviations. Discrete variables are presented as median and interquartile range (IQR). Categorical variables are reported using frequency and percentages. Four statistical analysis steps were taken in this study: descriptive statistical analyses, univariate analyses (i.e., simple linear regression and logistic regression), analyses of sociodemographic risk factors for prenatal depression and anxiety, and analyses of the relationship between prenatal depression and anxiety and birth outcomes (i.e., multivariable linear regression and logistic regression). T-tests and chi-square tests were conducted to compare characteristics between groups. The stepwise method was used to build the model. All statistical analyses were performed using STATA/BE 17.0. A p-value <0.05 was considered statistically significant.

### Ethics statement

This study was approved by the Research Ethics Boards of the University of Alberta (Pro00099276; Pro00087163), University of Calgary (Pro00099276_AME6; REB19-0336), York University (2020–117; 2018–179) and Anhui Medical University (2020H001). All participants were offered mental health resources and further counseling provided by the Mom’s Good Mood project [8].

## Results

### Characteristics of study population

A total of 3438 participants were recruited across the two study timepoints/cohorts. Of the 1189 participants recruited in the pre-pandemic cohort, 1148 (96.6%) participants responded to the survey. 2249 participants were recruited in the second cohort during the pandemic; all the 2249 provided a response to the survey. Although statistical tests showed significant differences between many variables, it is likely due to different samples size between the two. Otherwise, the two cohorts share similar distributions of demographic characteristics, with exception of household income and passive smoking (Table 1).

**Table 1:** Pre-pandemic and pandemic cohorts’ baseline characteristics.

Characteristics	Pre-pandemic (n = 1148)	Pandemic (n = 2249)	p-value
	n (%)	n (%)	
Age, mean (SD) (years)	28.2 (4.1)	28.4 (4.4)	0.199
BMI^a^, median (range) (kg/m^2^)	21.6 (3.2)	21.9 (3.4)	0.013
Socioeconomic status^b^, median (range)	5 (5–6)	6 (5–7)	<0.001
Number of people in the household, median (range)	3 (2–4)	3 (2–4)	0.001
Trimester			<0.001
First	1131 (95.1)	784 (34.9)	
Second	58 (4.9)	605 (26.9)	
Third	0	860 (38.2)	
Ethnicity			0.388
Han	1129 (98.8)	2210 (98.4)	
Other (Hui and other ethnicities)	14 (1.2)	36 (1.6)	
Residential area			0.001
Urban	1007 (87.7)	1549 (83.2)	
Rural	141 (12.3)	312 (16.8)	
Marital status			<0.001
Married	1075 (93.6)	1837 (99.1)	
Not married (divorced/separated/single/cohabit/widow)	73 (6.4)	17 (0.9)	
Household income (Yuan)			<0.001
<50,000	127 (11.1)	124 (7.1)	
50,000–100,000	436 (38.0)	440 (25.3)	
100,000–200,000	423 (36.9)	838 (48.2)	
200,000–300,000	104 (9.1)	241 (13.9)	
>300,000	58 (5.1)	94 (5.4)	
Education			0.017
Middle school or lower	198 (17.3)	356 (19.2)	
High school or equivalent	228 (19.9)	429 (23.1)	
Undergraduate or equivalent and higher	722 (62.9)	1070 (57.7)	
Employment			<0.001
Unemployed	479 (41.7)	910 (49.2)	
Employed (full-time/part-time/on paid leave)	669 (58.3)	941 (50.8)	
Smoking status			<0.001
Never smoke	1097 (95.6)	1824 (98.8)	
Used to or still smoking	51 (4.4)	23 (1.3)	
Passive smoking			<0.001
Never	359 (31.3)	1193 (65.1)	
Sometimes	593 (51.7)	488 (26.6)	
Almost everyday	196 (17.1)	152 (8.3)	
Alcohol consumption			<0.001
No^c^	993 (86.5)	1782 (97.2)	
Yes	155 (13.5)	52 (2.8)	
Attitude toward this pregnancy			0.310
Acceptable	549 (47.8)	864 (46.9)	
Unexpected	255 (22.2)	453 (24.6)	
Well-prepared	344 (30.0)	525 (28.5)	
Types of conception			<0.001
Non-assisted conception	1071 (93.3)	1780 (96.7)	
Assisted conception (IVF^d^/ICSI^e^/other)	77 (6.7)	61 (3.3)	
History of adverse pregnancy outcomes^f^			0.002
No	908 (79.1)	1495 (83.6)	
Yes	240 (20.9)	294 (16.4)	
History of mental health issues			0.024
No	1138 (99.1)	1803 (98.1)	
Yes	10 (0.9)	35 (1.9)	
History of using drugs			0.123
Never	1131 (98.5)	1817 (99.1)	
Used to or still use	17 (1.5)	16 (0.9)	
Multiparous			0.115
No	728 (63.4)	1363 (60.6)	
Yes	420 (36.6)	885 (39.4)	
Number of times being pregnant			0.738
First time pregnant	499 (43.5)	969 (43.1)	
Second time pregnant	338 (29.4)	691 (30.8)	
Third time pregnant	188 (16.4)	340 (15.1)	
Fourth time and above	123 (10.7)	247 (11.0)	

^a^ Body mass index.

^b^ Economic status compared to people around the participant, with 0 as the lowest and 10 the highest.

^c^ No alcohol consumption is defined as not more than one drink of either 340ml of beer or 140ml of wine or 43ml of Chinese Baijiu.

^d^ In vitro fertilization.

^e^ Intracytoplasmic sperm injection.

^f^ Adverse pregnancy outcomes include miscarriage, stillbirth, perinatal mortality, and birth defects.

#### Prenatal depression and anxiety

The pandemic cohort was found to have significantly lower mean EPDS scores compared to the pre-pandemic cohort (5.4 vs 5.8) (Table 2). Furthermore, the prevalence of prenatal depression symptoms (i.e., EPDS score ≥9) in the pandemic cohort was 14.5%, which was 4.1% lower than the prevalence (18.6%) in the pre-pandemic cohort. As for prenatal anxiety, a similar trend appeared. The mean GAD-7 scores were significantly higher in the pre-pandemic cohort than it was in the pandemic cohort (3.8 vs 2.9). The prevalence of prenatal anxiety measured by the GAD-7 (i.e., GAD-7 score≥5) was also higher in the pre-pandemic cohort (36.3%) than it was in the pandemic cohort (26.7%). Additionally, the rates of different severity levels of depression and anxiety symptoms were also lower in the pandemic cohort compared to the pre-pandemic cohort.

**Table 2:** Descriptive statistics of prenatal depression and anxiety for pre-pandemic (n = 1148) and pandemic (n = 2249) cohorts.

Characteristics	Pre-pandemic (n = 1148)	Pandemic (n = 2249)	p-value
	n(%)	n(%)	
EPDS^a^ scores, mean (SD^b^)	5.8 (3.4)	5.4 (3.3)	0.001
Prenatal Depression			0.002
No (0–8)	934 (81.4)	1923 (85.5)	
Yes (≥9)	214 (18.6)	326 (14.5)	
Severity of prenatal depression			0.020
No depression (0–8)	934 (81.4)	1923 (85.5)	
Mild depression (9–11)	142 (12.4)	220 (9.8)	
Moderate depression (12–13)	38 (3.3)	56 (2.5)	
Severe depression (≥14)	34 (3.0)	50 (2.2)	
GAD-7^c^ scores, mean (SD)	3.8 (3.4)	2.9 (2.9)	<0.001
Prenatal Anxiety			<0.001
No (0–4)	731 (63.7)	1649 (73.3)	
Yes (≥5)	417 (36.3)	600 (26.7)	
Severity of prenatal anxiety			<0.001
No anxiety (0–4)	731 (63.7)	1649 (73.3)	
Mild anxiety (5–9)	358 (31.2)	550 (24.5)	
Moderate anxiety (10–14)	44 (3.8)	40 (1.8)	
Severe anxiety (15–21)	15 (1.3)	10 (0.4)	

^a^ Edinburg Postnatal Depression Scale.

^b^ Standard deviation.

^c^ General Anxiety Disorder-7.

#### Risk factors of prenatal depression and anxiety

Pre-pandemic, we found that higher socioeconomic status was significantly associated with lower mean EPDS scores (β = -0.30, 95% CI: -0.44 to -0.17, *p*<0.001). This indicates that participants with higher incomes experienced less severe depression symptoms, compared to people with lower socioeconomic status in the multivariable model. Participants who reported having more people in the household, not being married, formerly smoked, or were still smoking, or had an unexpected pregnancy at the time were found to be at greater risk of experiencing higher levels of depressive symptoms (Table 3). As for prenatal anxiety, we found that older pregnant women had lower GAD-7 scores (β = -0.05, 95% CI: -0.1, -0.004, *p* = 0.031) in the pre-pandemic cohort. Participants who were not married, consuming alcohol, exposed to passive smoking, had an unexpected pregnancy, and reported a prior history of adverse pregnancy outcomes were likely to have higher GAD-7 scores (Table 3).

**Table 3:** Risk factors of prenatal depression and prenatal anxiety among the pre-pandemic cohort (n = 1148)–multivariable analysis.

Characteristics	Prenatal Depression (EPDS score)		Prenatal Anxiety (GAD-7 score)	
	Coef. (95%CI^a^)	p-value	Coef. (95%CI^a^)	p-value
Age (years)			-0.05 (-0.10, -0.005)	0.031
Socioeconomic status^b^	-0.30 (-0.44, -0.17)	<0.001		
Number of people in the household	0.27 (0.11, 0.44)	0.001		
Marital status				
Married	Reference		Reference	
Not married (divorced/separated/ single/cohabit/widow)	1.27 (0.46, 2.08)	0.002	1.03 (0.21, 1.84)	0.014
Smoking status				
Never smoke	Reference			
Used to or still smoking	1.03 (0.09, 1.97)	0.032		
Passive smoking				
Never			Reference	
Sometimes			0.66 (0.23, 1.10)	0.003
Almost everyday			0.96 (0.38, 1.54)	0.001
Alcohol consumption				
No^c^			Reference	
Yes			0.75 (0.18, 1.32)	0.010
Attitude toward this pregnancy				
Acceptable	Reference		Reference	
Unexpected	0.93 (0.43, 1.43)	<0.001	0.51 (0.01, 1.02)	0.045
Well-prepared	-0.12 (-0.57, 0.34)	0.607	0.09 (-0.36, 0.54)	0.691
History of adverse pregnancy outcomes^d^				
No			Reference	
Yes			0.52 (0.04, 1.00)	0.034

^a^ 95% confidence interval.

^b^ Economic status compared to people around the participant, with 0 as the lowest and 10 the highest.

^c^ No alcohol consumption is defined as not more than one drink of either 340ml of beer or 140ml of wine or 43ml of Chinese Baijiu.

^d^ Adverse pregnancy outcomes include miscarriage, stillbirth, perinatal mortality, and birth defects.

In the pandemic cohort, we found that women in their second or third trimesters, with higher socioeconomic statuses and who were of older ages, had lower mean EPDS scores in the multivariable model (Table 4). On the other hand, exposure to passive smoking, having a history of alcohol consumption, a history of mental health issues, higher education levels, reporting an unexpected pregnancy, and more people in the household were found to be risk factors associated with higher EPDS scores. Results showed that women who reported drinking had higher overall mean EPDS scores compared to women who did not drink. Furthermore, we found a significant interaction between trimester and alcohol consumption. Mean EPDS scores, among women who reported drinking, were highest in the first trimester compared to in the second and third trimesters. As for prenatal anxiety, results showed that women who were in their second or third trimester, who reported higher socioeconomic status and were older, had lower mean GAD-7 scores. Exposure to passive smoking, drinking alcohol, prior adverse pregnancy outcomes, history of mental health issues, and greater household size were risk factors for higher mean GAD-7 scores.

**Table 4:** Risk factors of prenatal depression and prenatal anxiety among the pandemic cohort (n = 2249)–multivariable analysis.

Characteristics	Prenatal Depression (EPDS score)		Prenatal Anxiety (GAD-7 score)	
	Coef. (95%CI^a^)	p-value	Coef. (95%CI^a^)	p-value
Age (years)	-0.10 (-0.11, -0.04)	<0.001	-0.06 (-0.09, -0.03)	<0.001
Socioeconomic status^b^	-0.22 (-0.32, -0.12)	<0.001	-0.14 (-0.23, -0.05)	0.002
Number of people in the household	0.22 (0.09, 0.35)	0.001	0.13 (0.02, 0.24)	0.021
Trimester				
First	Reference		Reference	
Second	-0.54 (-0.94, -0.13)	0.009	-1.09 (-1.44, -0.75)	<0.001
Third	-0.41 (-0.77, -0.05)	0.027	-0.85 (-1.16, -0.54)	<0.001
Education				
Middle school or lower	Reference			
High school or equivalent	-0.68 (-1.16, -0.19)	0.006		
Undergraduate or equivalent and higher	-0.65 (-1.08, -0.23)	0.003		
Passive smoking				
Never	Reference		Reference	
Sometimes	0.28 (-0.07, 0.63)	0.114	0.28 (-0.02, 0.59)	0.072
Almost everyday	0.79 (0.21, 1.37)	0.008	0.63 (0.13, 1.14)	0.014
Alcohol consumption				
No^c^	Reference		Reference	
Yes	3.66 (2.05, 5.28)	<0.001	0.83 (0.04, 1.61)	0.039
Attitude toward this pregnancy				
Acceptable	Reference			
Unexpected	0.46 (0.07, 0.84)	0.020		
Well-prepared	-0.04 (-0.40, 0.32)	0.837		
History of adverse pregnancy outcomes^d^				
No			Reference	
Yes			0.40 (0.04, 0.77)	0.031
History of mental health issues				
No	Reference		Reference	
Yes	2.85 (1.73, 3.97)	<0.001	2.91 (1.93, 3.89)	<0.001
Interactions				
Trimester*Alcohol Consumption				
Second trimester*Yes	-2.99 (-5.14, -0.84)	0.006		
Third trimester*Yes	-3.28 (-5.65, -0.92)	0.006		

^a^ 95% confidence interval.

^b^ Economic status compared to people around the participant, with 0 as the lowest and 10 the highest.

^c^ No alcohol consumption is defined as not more than one drink of either 340ml of beer or 140ml of wine or 43ml of Chinese Baijiu.

^d^ Adverse pregnancy outcomes include miscarriage, stillbirth, perinatal mortality, and birth defects.

#### Adverse birth outcomes

There was no significant difference in the mean birth weight between pre-pandemic and pandemic cohorts. Although the rate of low birth weight was slightly lower in the pandemic cohort, and the rate of small for gestational age was slightly higher in the pandemic cohort, the differences was not significant (Table 5).

**Table 5:** Descriptive statistics of birth outcomes for pre-pandemic (n = 1148) and pandemic (n = 2249) cohorts.

Characteristics	Pre-pandemic (n = 1148)	Pandemic (n = 2249)	p-value
	n (%)	N (%)	
Birth weight, mean (SD) (grams)	3371 (470)	3374 (454)	0.879
Gestation weeks, mean (SD) (weeks)	39.5 (1.6)	39.5 (1.5)	1.000
Low birth weight			0.493
No (≥2500g)	1080 (96.8)	2186 (97.2)	
Yes (<2500g)	36 (3.2)	63 (2.8)	
Severity of low birth weight			0.452
No (≥2500g)	1080 (96.8)	2186 (97.2)	
Moderate low birth weight (1500g – 2500g)	35 (3.1)	58 (2.6)	
Very low birth weight (1000g – 1500g)	1 (0.1)	5 (0.2)	
Preterm birth			0.979
No (≥37 weeks)	1070 (95.7)	2152 (95.7)	
vYes (<37 weeks)	48 (4.3)	97 (4.3)	
Severity of preterm birth			0.133
No (≥37 weeks)	1070 (95.7)	2152 (95.7)	
Moderate to late preterm birth (32–37 weeks)	40 (3.6)	91 (4.1)	
Very preterm birth (28–32 weeks)	8 (0.7)	6 (0.3)	
Small for gestational age			0.607
No	1036 (93.8)	1952 (94.2)	
Yes	69 (6.2)	120 (5.8)	

#### Association between prenatal mental health issues and adverse birth outcomes

In the pre-pandemic cohort, birth weight, gestational age, and small for gestational age were not significantly associated with prenatal depression or anxiety (Tables 6 and 7). However, participants’ pre-pregnancy BMI, marital status, household income, and types of conception were significantly associated with infants’ birth weight in the multivariable model. For the gestational age outcome, participants’ age, types of conception, and multi-parity were statistically significant in the adjusted model. Participants’ pre-pregnancy BMI, number of people in the household, education, and multi-parity were significantly associated with small for gestational age.

**Table 6:** Association between prenatal depression and birth outcomes among the pre-pandemic cohort—multivariable models.

Characteristics	Birth weight (grams)		Gestational age (weeks)		Small for gestational age	
	Coef. (95%CI^a^)	p-value	Coef. (95%CI^a^)	p-value	OR^b^ (95%CI^a^)	p-value
EPDS score	-3.68 (-12.24, 4.87)	0.398	-0.02 (-0.05, 0.003)	0.082	0.97 (0.90, 1.05)	0.435
Age (years)			-0.03 (-0.05, -0.003)	0.030		
BMI^c^ (kg/m^2^)	16.26 (7.45, 25.06)	<0.001			0.90 (0.82, 0.98)	0.020
Marital status						
Married	Reference					
Not married (divorced/separated/ single/cohabit/widow)	124.99 (9.42, 240.57)	0.034				
Household income (Yuan)						
<50,000	Reference					
50,000–100,000	146.34 (50.60, 242.08)	0.003				
100,000–200,000	102.36 (6.29, 198.44)	0.037				
200,000–300,000	166.30 (40.32, 292.29)	0.010				
>300,000	173.15 (22.67, 323.64)	0.024				
Education						
Middle school or lower					Reference	
High school or equivalent					0.60 (0.28, 1.30)	0.196
Undergraduate or equivalent and higher					0.42 (0.22, 0.81)	0.010
Types of conception						
Non-assisted conception	Reference		Reference			
Assisted conception (IVF^d^/ICSI^e^/other)	-156.08 (-266.7, -45.42)	0.006	-1.08 (-1.45, -0.70)	<0.001		
Multiparous						
No			Reference		Reference	
Yes			-0.42 (-0.64, -0.20)	<0.001	0.53 (0.30, 0.96)	0.037

^a^ 95% confidence interval.

^b^ Odds ratio.

^c^ Body mass index.

^d^ In vitro fertilization.

^e^ Intracytoplasmic sperm injection.

**Table 7:** Association between prenatal anxiety and birth outcomes among the pre-pandemic cohort—multivariable models.

Characteristics	Birth weight (grams)		Gestational age (weeks)		Small for gestational age	
	Coef. (95%CI^a^)	p-value	Coef. (95%CI^a^)	p-value	OR^b^ (95%CI^a^)	p-value
GAD-7 score	-3.26 (-11.83, 5.30)	0.455	-0.02 (-0.05, 0.01)	0.207	0.95 (0.87, 1.03)	0.214
Age (years)			-0.03 (-0.05, -0.002)	0.035		
BMI^c^ (kg/m^2^)	16.25 (7.45, 25.06)	<0.001			0.90 (0.82, 0.98)	0.019
Marital status						
Married	Reference					
Not married (divorced/separated/ single/co-habit/widow)	123.91 (8.44, 239.37)	0.035				
Household income (Yuan)						
<50,000	Reference					
50,000–100,000	148.88 (53.35, 244.42)	0.002				
100,000–200,000	105.68 (10.00, 201.36)	0.030				
200,000–300,000	170.43 (44.46, 296.39)	0.008				
>300,000	178.44 (28.68, 328.21)	0.020				
Education						
Middle school or lower					Reference	
High school or equivalent					0.60 (0.28, 1.30)	0.198
Undergraduate or equivalent and higher					0.44 (0.23, 0.84)	0.013
Types of conception						
Non-assisted conception	Reference		Reference			
Assisted conception (IVF^d^/ICSI^e^/other)	-155.48 (-266.12, -44.84)	0.006	-1.08 (-1.45, -0.70)	<0.001		
Multiparous						
No			Reference		Reference	
Yes			-0.44 (-0.66, -0.22)	<0.001	0.5 (0.3, 0.9)	0.033

^a^ 95% confidence interval.

^b^ Odds ratio.

^c^ Body mass index.

^d^ In vitro fertilization.

^e^ Intracytoplasmic sperm injection.

Overall, results collected during the pandemic showed that prenatal depression was associated with a higher risk of having small for gestational age infants, with the key interactions between trimester and EPDS, in the model (Table 8). Women who were in their first trimester, with higher EPDS scores, were at the highest risk of giving birth to infants with small for gestational age weight, compared to women in the second and third trimesters. Women who are in their third trimester, with higher EPDS scores, had the lowest risk of small for gestational age, compared to women in the other two trimesters. Prenatal depression was not significantly associated with low birth weight or preterm birth. Prenatal anxiety was also not associated with any adverse birth outcomes, in this study (Table 9).

**Table 8:** Association between prenatal depression and birth outcomes among the pandemic cohort—multivariable models.

Characteristics	Birth weight (grams)		Gestational age (weeks)		Small for gestational age	
	Coef. (95%CI^a^)	p-value	Coef. (95%CI^a^)	p-value	OR^b^ (95%CI^a^)	p-value
EPDS score	4.68 (-1.21, 10.56)	0.120	0.01 (-0.01, 0.03)	0.236	1.09 (1.00, 1.19)	0.042
Age (years)			-0.02 (-0.04, -0.004)	0.014		
BMI^c^ (kg/m^2^)	11.97 (6.21, 17.73)	<0.001				
Trimester						
First			Reference		Reference	
Second			-0.21 (-0.37, -0.04)	0.012	1.62 (0.65, 4.09)	0.303
Third			0.06 (-0.09, 0.20)	0.443	2.30 (0.99, 5.36)	0.054
Multiparous						
No	Reference		Reference		Reference	
Yes	66.17 (26.21, 106.12)	0.001	-0.17 (-0.32, -0.03)	0.012	0.61 (0.41, 0.91)	0.016
Interaction						
Trimester*EPDS score						
Second trimester					0.95 (0.83, 1.09)	0.503
Third trimester					0.86 (0.76, 0.98)	0.026

^a^ 95% confidence interval.

^b^ Odds ratio.

^c^ Body mass index.

**Table 9:** Association between prenatal anxiety and birth outcomes among the pandemic cohort—multivariable models.

Characteristics	Birth weight (grams)		Gestational age (weeks)		Small for gestational age	
	Coef. (95%CI^a^)	p-value	Coef. (95%CI^a^)	p-value	OR^b^ (95%CI^a^)	p-value
GAD-7 score	3.35 (-3.46, 10.17)	0.335	0.01 (-0.01, 0.03)	0.407	1.02 (0.96, 1.08)	0.582
Age (years)			-0.02 (-0.04, -0.005)	0.011		
BMI^c^ (kg/m^2^)	12.09 (6.33, 17.85)	<0.001				
Trimester						
First			Reference			
Second			-0.20 (-0.37, -0.04)	0.015		
Third			0.06 (-0.09, 0.21)	0.419		
Multiparous						
No	Reference		Reference		Reference	
Yes	67.07 (27.03, 107.11)	0.001	-0.17 (-0.31, -0.02)	0.023	0.62 (0.41, 0.93)	0.021

^a^ 95% confidence interval.

^b^ Odds ratio.

^c^ Body mass index.

Multiparous was significantly associated with infants’ birth weight, gestational age and small for gestational age. Participants’ who had higher pre-pregnancy BMI was associated with higher infants’ birth weight. Participants with older age was associated with lower infants’ gestational age (Tables 8 and 9).

## Discussion

### Summary of findings

This study assessed the prevalence of prenatal depression, anxiety, low birth weight, preterm birth, and small for gestational age weight. We also identified potential sociodemographic risk factors for these mental health and birth outcomes before and during the COVID-19 pandemic in Ma’anshan, Anhui, China, as well as explored the relationship between prenatal mental health and birth outcomes. We found that the prevalence of prenatal depression and anxiety was lower during the pandemic compared to in the pre-pandemic period. The prevalence of each adverse birth outcome was not significantly different between the two cohorts, although the prevalence of low birth weight and small for gestational age was slightly lower during the pandemic. Furthermore, we only found prenatal depression was significantly associated with higher risk of small for gestational age during the pandemic period. Risk factors for prenatal depression and anxiety varied between pre-pandemic and pandemic periods.

### Prenatal depression and anxiety

Compared to studies conducted before the COVID-19 pandemic using the same/similar screening tools, prenatal depression prevalence (18.6%) measured in this study was like that reported in another study also conducted in Anhui (19.1%) [21,30]. Prenatal anxiety prevalence in our study (36.3%) before the pandemic was almost the same as reported in the cities of Shenyang, Zhengzhou, and Chongqing (36.4%) [31]. During the pandemic, prenatal depression prevalence was 14.5%, which was much lower than was reported in Wuhan (33.7%), the epicenter of the COVID-19 outbreak in China [23]. The prevalence of prenatal anxiety during the pandemic, in our study, was 26.7%, which was different than in other parts of China, including Changzhou (31.7%), Guangzhou (19.0%), and Shenyang (11.8%) [32–34]. Differences in depression and anxiety prevalence during the pandemic could be due to the localized severity of the pandemic across multiple cities [35], as well as the use of different screening tool cut-off points [36].

An interesting finding was that the prevalence of prenatal depression and anxiety was lower in the pandemic cohort than it was in the pre-pandemic cohort. Many published studies reported higher rates of prenatal depression and anxiety during the pandemic compared to pre-pandemic periods. This is possibly due to the uncertainty regarding the effects of COVID-19 on fetal health, pandemic restrictions, and pandemic-related income loss [4,6,7,37]. There were fewer studies that reported lower rates of prenatal mental health issues [38,39]. Another longitudinal study conducted in China reported non-significant differences in anxiety and depression between pandemic and pre-pandemic periods [40]. However, this study focused on the general population, rather than pregnant women. In our study, the reduction in prenatal depression and anxiety rates could be due to different factors. First, women may be more likely to receive more family support and care during their pregnancies, given household structures in China, which could have been protective [1,41]. A study reported that increased social support increases people’s resilience to stress, further leading to decreased risk of developing prenatal anxiety and depression [42]. Second, social activities during the pandemic were limited in China. For instance, due to pandemic social restrictions, pregnant women no longer needed to go to work in-person, which may have translated into more time to focus on their pregnancy and family. Findings could also be due to the severity of the COVID-19 pandemic in Ma’anshan, which was lower compared to the cities examined in other studies that reported increased prenatal depression and anxiety [4,6,7,37]. Another study reported that women from the pandemic’s hardest-hit areas in China were more likely to experience anxiety [43]. In addition, China actively, quickly, and strictly implemented COVID-19 management and control strategies to minimize the impact of the pandemic on the economy, society, production, and peoples’ lives [44]. These strategies may have eased stress and panic. It is also important to note that differences may have arisen in this study as the pre-pandemic and pandemic cohorts did not include the same participants. Differences, other than those assessed in the study, might also have impacted these results. Further longitudinal research is needed to explore changes in the prevalence of prenatal depression and anxiety across time in the same population.

In our study, we also assessed the risk factors of prenatal depression and anxiety. Like many other studies that reported significant associations between prenatal depression and alcohol consumption [13–16], we also found that women who reported drinking had overall higher mean EPDS scores than those who did not drink. This could be due to that women who did not drink are less likely to stress about the consequences of drinking on their fetus, but the ones who reported drinking might also experience more stress since they may worry about their drinking behavior could have damaged the development of the baby. However, several studies have reported no association between alcohol use and prenatal depression [45]. The inconsistency in results could also be due to differences in measurement of depression and alcohol use. In addition, we only assessed general alcohol consumption status, rather than consumption during pregnancy. Thus, it is unclear if prenatal depression occurs after or prior to alcohol consumption. We also observed that mean EPDS scores were the highest among women in their first trimester compared to women in their second or third trimester. The first trimester is often a time of adjustment, which may lead to higher levels of stress. It could also be due to women in their first trimester often experiencing discomfort or severe pregnancy sickness, such as nausea.

### Adverse birth outcomes and association with prenatal mental health issues

To our knowledge, this is the first study that explored and compared the impact of prenatal depression and anxiety on birth outcomes in China before and during the COVID-19 pandemic. Previous studies have largely been conducted before the pandemic or during the pandemic, but not in both time periods [16]. Although some findings are inconsistent with prior results, this study contributes to the literature around potential pathways and impacts of prenatal depression and anxiety on birth outcomes, through the comparison of stress before and during a public health emergency.

In this study, we found that prenatal depression or anxiety was not significantly associated with lower birth weight, lower gestational age or small for gestational age in the pre-pandemic period after adjusting for other covariates. Similarly, many studies reported that prenatal depression or anxiety was not associated with an increased risk of low birth weight or decreases in birth weight, preterm birth, or small for gestational age [30,46–48]. However, in the pandemic cohort, small for gestational age was significantly associated with prenatal depression. This could be due to the imbalanced neuroendocrine caused by prenatal depression that has impacted on the fetal development. Experts hypothesize that depression during pregnancy may stimulate the hypothalamus-pituitary-adrenal (HPA) system and lead to hypo- or hypersecretion of cortisol and norepinephrine, decreasing uterine blood flow, leading to parturition, and impaired fetal development and growth [20,21,49]. Similar findings on the association between small for gestational age and prenatal depression were also reported in other studies [30,50–58].

### Limitations

There are some limitations to this study. First, due to the nature of the data, this study only focused on exploring the association between prenatal mental health and birth outcomes, rather than a causal relationship between the two. Second, the pre-pandemic and pandemic cohorts differed in size. This is due to the fact that pre-pandemic data collection was part of another project. Separate sample size estimations were calculated for the pandemic cohort to explore prenatal depression and anxiety in relation to low birth weight, preterm birth, and small for gestational age weight. Women were recruited from the same region to ensure comparability, though this might affect the generalizability of these results. Third, prenatal depression and anxiety were assessed online using self-reported questionnaires. Although these tools have been demonstrated to have good reliability and validity in the Chinese population, the potential for reporting bias cannot be overlooked. The inclusion of clinical diagnoses might help to reduce this bias in future studies.

## Conclusion

In this study, the prevalence of prenatal depression, anxiety and adverse birth outcomes was lower after the onset of the COVID-19 pandemic. A significant association between prenatal depression and small for gestational age during the pandemic was found. More research on prenatal depression and anxiety trends during the pandemic, as well as their impacts on birth outcomes, is needed. This type of research can help inform national plans, establish health priorities, and guide clinical and public health responses to COVID-19 and other future pandemics and disasters.
